# Navigating Complex Challenges: Preoperative Assessment and Surgical Strategies for Liver Resection in Patients with Fibrosis or Cirrhosis

**DOI:** 10.3390/biomedicines12061264

**Published:** 2024-06-06

**Authors:** Jennifer A. Kalil, Marc Deschenes, Hugo Perrier, Oran Zlotnik, Peter Metrakos

**Affiliations:** 1 Department of Surgery, Royal Victoria Hospital, McGill University Health Center, 1001 Blvd Decarie, Montreal, QC H4A 3J1, Canada; jennifer.kalil@mail.mcgill.ca (J.A.K.); hugo.perrier@mail.mcgill.ca (H.P.); oran.zlotnik@mail.mcgill.ca (O.Z.); 2 Cancer Research Program, McGill University Health Center, Research Institute, 1001 Blvd Decarie, Montreal, QC H4A 3J1, Canada; 3 Department of Medicine, Division of Gastroenterology & Hepatology & Transplantation, Royal Victoria Hospital, McGill University Health Center, 1001 Blvd Decarie, Montreal, QC H4A 3J1, Canada; marc.deschenes@mcgill.ca

**Keywords:** cirrhosis, liver resection, surgical risk assessment, portal hypertension, hepatocellular carcinoma

## Abstract

This review explores the intricacies of evaluating cirrhotic patients for liver resection while exploring how to extend surgical intervention to those typically excluded by the Barcelona Clinic Liver Cancer (BCLC) criteria guidelines by focusing on the need for robust preoperative assessment and innovative surgical strategies. Cirrhosis presents unique challenges and complicates liver resection due to the altered physiology of the liver, portal hypertension, and liver decompensation. The primary objective of this review is to discuss the current approaches in assessing the suitability of cirrhotic patients for liver resection and aims to identify which patients outside of the BCLC criteria can safely undergo liver resection by highlighting emerging strategies that can improve surgical safety and outcomes.

## 1. Introduction

**Figure 1:**
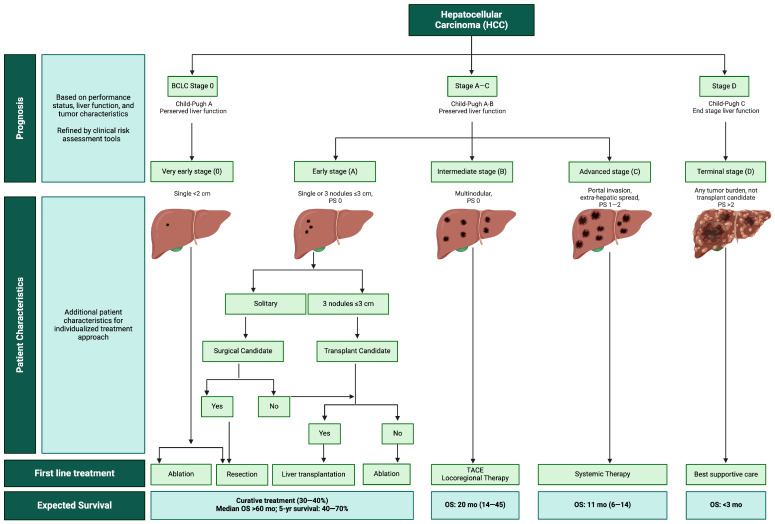
Barcelona Clinic Liver Cancer (BCLC) staging and treatment strategy algorithm. PS, performance status; TACE, transarterial chemoembolization; OS, overall survival; yr, year; mo, months.

The most frequent indication for liver resection in patients with cirrhosis is hepatocellular carcinoma (HCC) [1]. HCC is the sixth most common cancer and the fourth leading cause of cancer-related deaths worldwide [2]. In 90% of cases, HCC arises in the background of a cirrhotic liver, making the treatment options complex [3]. Staging classification systems that focus on either tumor stage or severity of liver disease lack prognostic accuracy. The Barcelona Clinic Liver Cancer (BCLC) staging and treatment strategy has become the most widely used clinical tool for treatment decisions as it integrates tumor stage, liver function, and performance status into the treatment algorithm (Figure 1) [4]. In early-stage disease, the algorithm advocates for potentially curative treatments such as resection, ablation, or liver transplantation, based on stringent criteria that consider the tumor size, tumor number, and absence of vascular invasion [4]. For patients with intermediate-stage HCC, the BCLC algorithm recommends transarterial chemoembolization (TACE), and in patients with advanced disease, the management has evolved to incorporate novel targeted therapy and immunotherapies [5].

While the BCLC algorithm is widely recognized and utilized in the management of HCC, the indications for liver resection are quite restrictive, which may not account for individual patient variations or the nuanced clinical judgment required in complex cases. Although resection is often regarded as the most effective treatment for HCC, it is not recommended for patients with large, multinodular HCC or those presenting with macrovascular invasion [6]. Additionally, even when tumors are of optimal size and number, resection is relatively contraindicated in patients with portal hypertension. Studies have recently challenged this paradigm and show promising results after surgical resection in patients beyond the BCLC criteria, leading to a growing interest in expanding the indication for liver resection outside BCLC guidelines [6,7].

The objective of this review is to explore the potential for safely expanding operative criteria while investigating the optimization of patients beyond the BCLC criteria for liver resection and to identify predictive factors indicative of surgical success or failure.

## 2. Pathophysiology of Cirrhosis and Portal Hypertension

### 2.1. Pathogenesis of Cirrhosis

One of the most significant complications of cirrhosis is portal hypertension, which is one of the contraindications to liver resection according to the BCLC treatment strategy. Cirrhosis results from an underlying liver parenchymal injury that leads to both inflammation and cellular necrosis with fibrogenesis. Inflammatory cascades lead to the activation of hepatic stellate cells, which promote extracellular matrix deposition and fibrogenesis. This causes vascular occlusion with the collapse of liver structures, loss of parenchymal cells, and intrahepatic shunting. Microvascular changes and angiogenesis occur with sinusoidal remodeling, causing a marked architectural distortion and resulting in increased resistance to portal venous flow [4,8]. Hepatocyte perfusion is decreased due to the changes in portal flow and intrahepatic shunting, which contributes to worsening liver function.

### 2.2. Portal Hypertension

Portal hypertension (PH) is characterized by increased pressure in the portal venous system and has long been considered a contraindication to liver resection. PH is most frequently associated with cirrhosis and develops due to increased resistance in portal flow [9]. Clinical manifestations of PH include varices, thrombocytopenia, splenomegaly, ascites, and evidence of portosystemic collaterals on radiographic imaging [10]. Clinically significant portal hypertension (CSPH) develops when the hepatic venous pressure gradient (HVPG) is >10 mmHg and is the precursor to hepatic decompensation, defined as the onset of complications from cirrhosis including ascites, hepatic encephalopathy, variceal bleeding, and hepatorenal syndrome [9]. Many studies have demonstrated that patients with CSPH who undergo liver resection are more likely to develop liver decompensation postoperatively [11,12].

Portal hypertension can manifest in the absence of cirrhosis and its associated parenchymal alterations. Noncirrhotic portal hypertension, or porto-sinusoidal vascular disorder, is broadly categorized as pre-hepatic (portal or splenic vein thrombosis), post-hepatic (Budd-Chiari syndrome), intrahepatic (presinusoidal, sinusoidal, or post-sinusoidal), and idiopathic [10,13]. Although these patients may present with signs and symptoms of portal hypertension, they typically do not exhibit an elevated HVPG or results on transient elastography (Fibroscan) to suggest increased liver stiffness [14].

## 3. Clinical Significance

### Surgical Complications Associated with Cirrhosis and Portal Hypertension

Liver failure and cirrhosis pose significant challenges to perioperative management, affecting multiple organ systems. Patients with cirrhosis commonly experience protein synthesis dysfunction and malnutrition, increasing the risk of postoperative complications including impaired wound healing, infections, and ascites [15]. Ascites development further exacerbates complications, leading to increased infection, wound complications, and increased risk of postoperative renal failure.

Cirrhotic patients exhibit “rebalanced hemostasis” due to changes in the coagulation pathways that favor both clotting and bleeding because of decreased procoagulant and anticoagulant proteins [16]. However, these patients face an elevated risk of bleeding with surgical procedures mostly due to fluid shifts leading to variceal bleeds [17]. Fluid management in cirrhotic patients can be difficult; excessive fluid can cause variceal bleeding, while inadequate fluid may result in hypotension and relative hepatic ischemia.

These factors collectively contribute to increased postoperative complications and the risk of hepatic decompensation. Liver resection in cirrhotic patients portends an even higher risk of hepatic decompensation by reducing remnant liver mass and has led to the widely accepted recommendation to limit or even avoid liver resection in these patients altogether [17].

## 4. Estimating Surgical Risk

Despite notable advancements in the surgical techniques and perioperative care of patients undergoing hepatic resection, the management of patients with chronic liver disease, cirrhosis, and/or portal hypertension remains challenging, with an increased risk of perioperative morbidity and mortality [18,19]. The degree of liver disease directly correlates with surgical risk, with the Child–Pugh score serving as a longstanding primary tool for prognostic assessment in cirrhotic patients for over four decades [20]. Additionally, several clinically valuable risk models have been developed to predict perioperative risk in this patient population.

### 4.1. Child–Turcotte–Pugh Classification (CTP)

The CTP score, originally developed to guide the selection of patients who would benefit from elective portosystemic shunt surgery, is widely used to predict mortality after surgery in cirrhotic patients [21]. It is based on both clinical and subjective factors (presence and severity of ascites and encephalopathy) as well as objective tests (international normalized ratio, albumin, and bilirubin), which reflect the degree of hepatic synthetic dysfunction [22]. Each variable is assigned 1–3 points, and the aggregate score categorizes the degree of cirrhosis into three classes: class A (5–6 points) represents well-compensated cirrhosis, class B (7–9 points) represents mild decompensated cirrhosis, and class C (10–15 points) represents severe decompensated cirrhosis [23]. The predicted mortality after elective surgery associated with CTP class A, B, and C is 10%, 30%, and 80%, respectively [23].

### 4.2. Model for End-Stage Liver Disease (MELD)

The MELD score was originally developed to predict mortality after transjugular intrahepatic portosystemic shunt (TIPS) to determine the patients who were likely to progress to requiring liver transplantation [24]. It is derived from a mathematical model using INR, serum creatinine, and serum bilirubin. The score ranges from 6 to 40, with higher scores conveying a worse prognosis and higher mortality [25]. The MELD correlates well with CTP in predicting mortality after elective surgery in cirrhotic patients. Because of the model’s objectivity and the ability to accurately predict short-term survival, the MELD score was widely adopted to prioritize allocation of organs for liver transplantation [21].

### 4.3. Mayo Risk Score (MRS)

The MRS was designed to predict both short- and long-term mortality after elective surgery in patients with cirrhosis. This score combines the MELD score, the American Society of Anesthesiologists (ASA) physical status class, and the patient’s age [19]. A study involving 772 cirrhotic patients who underwent major abdominal, orthopedic, or cardiovascular surgery revealed that ASA class V significantly predicted mortality within the initial 7 days [19]. In contrast, the MELD score was the most reliable predictor of mortality beyond 7 days and demonstrated a linear correlation with mortality risks [19].

Incorporating the patient’s age and ASA status, the MRS encompasses the individual’s overall physical condition, rendering it a more complete evaluation of surgical risk and allowing improved preoperative assessment and enhanced surgical planning. However, by incorporating multiple variables and other risk models (i.e., MELD) into the score, the complexity of the score increases. The MRS predicts both short-term and long-term mortality, making it a versatile tool across various types of surgeries. Yet, lack of consideration for specific types of surgery may limit the precision in risk stratification for those procedures [19].

### 4.4. Veterans Outcomes and Costs Associated with Liver Disease (VOCAL-Penn) Model

VOCAL-Penn is a recent surgical risk prediction model designed to more accurately predict postoperative mortality while considering specific operations. Unlike previous models, it stratifies risk according to the type of surgery, thereby providing a more tailored risk assessment [26]. This model stratifies patients’ risk of mortality at 30, 90, and 180 days postoperatively using a model derived from patient age, albumin, total bilirubin, platelet count, body mass index (BMI), presence of non-alcoholic fatty liver disease (NAFLD), ASA, elective vs. emergent, and type of surgery [26]. This risk prediction model incorporates a comprehensive set of patient variables in addition to the surgery type, resulting in a detailed risk profile and enhancing the predictive accuracy [26]. Highlighting this, the VOCAL-Penn study found that the calibration of the MRS decreased over time, resulting in consistent overprediction of mortality risk. Additionally, the type of surgery was identified as a critical predictor of mortality risk [26].

While the comprehensive nature of the model, including more clinical variables, enhances its predictive accuracy within the original study cohort, it also increases the risk of overfitting, making it less generalizable to populations outside the original study cohort. Additionally, there may have been factors contributing to patient selection for surgery that could not be accounted for in the development of the model and will likely need periodic recalibration. Finally, although the model considers a wide breadth of operations, liver resections were excluded [26].

### 4.5. Fibrosis-4 (FIB-4)

FIB-4 is a clinical tool used primarily in patients with NAFLD to assess liver fibrosis [27]. The score is generated from patient age, aspartate aminotransferase (AST), alanine aminotransferase (ALT), and platelet count and is calculated using the following equation:

$$\mathrm{FIB}\text{-}4=\frac{Age\;(years)\times AST\;(U/L)}{Platelet\;count\;(10^{9}/L)\times \surd ALT\;(U/L)}$$

The FIB-4 score stratifies patients into validated categories of low risk or ruling out advanced stage fibrosis (score of ≤ 1.3), inconclusive (score of > 1.3 and < 2.67), and suggestive of advanced stage fibrosis (score or ≥ 2.67). It is particularly useful in patients without overt signs of liver disease, as advanced fibrosis can often go undiagnosed [28]. One study demonstrated that elevated FIB-4 scores in patients without apparent liver disease conveyed two-fold increased mortality intra-operatively, during hospitalization, and within 30 days of surgery [28].

### 4.6. Forns Score

The Forns score is a non-invasive tool developed to identify patients with and without significant liver fibrosis (stage 2–4 versus stage 0–1) in those with chronic hepatitis C to potentially avoid liver biopsy in a subset of individuals [29]. A mathematical model was designed from four clinical variables, including patient age, gamma-glutamyl transferase (GGT), cholesterol levels, and platelet count, to calculate a score from the following equation:

7.811 − 3.131 × ln[platelet count (10^9^/L)] + 0.781 × ln[GGT (U/L)] + 3.467 × ln(age) − 0.014 × [total cholesterol (U/L)]

The score is used to identify the presence (score of > 6.9) or absence (score of < 4.21) of significant fibrosis. The model demonstrated a high negative predictive value with a score below 4.2, identifying patients without significant fibrosis with 96% certainty, while the positive predictive value for a score > 6.9 was only 44% [29].

### 4.7. Albumin-Bilirubin Score (ALBI)

The ALBI score was created to predict overall survival after hepatectomy in a cohort of patients with HCC. It is derived from serum bilirubin (umol/L) and albumin (g/L) and calculated using the following equation:

ALBI = [log10 bilirubin (umol/L) × 0.66] + [albumin (g/L) × −0.085]

The ALBI score stratifies patients into three grades: grade 1 (≤−2.6), grade 2 (−2.6 to −1.39), and grade 3 (>−1.39), where grades 2 and 3 are classified as high-risk [30]. This model was also studied in comparison to the MELD and was found to be a more accurate predictor of post-hepatectomy liver failure (PHLF) and postoperative mortality [30].

### 4.8. Combined Aspartate Aminotransferase/Platelet Ratio Index (APRI)/ALBI

The APRI score was initially developed to assess liver fibrosis in patients with chronic hepatitis C [31]. Like the Forns score, it was created as a non-invasive alternative to liver biopsy for evaluating the extent of hepatic fibrosis and cirrhosis [31]. The score is derived from AST and platelet count using the following formula:

$$APRI=\frac{\left[AST\;(U/L)]/[AST\;(upper\;limit\;normal)\right]}{Platelet\;count\;(10^{9}/L)}\times 100$$

The thresholds for detecting significant fibrosis and cirrhosis were 0.7 and 1, respectively [31]. More recent studies demonstrated that the combination of the APRI/ALBI scores was predictive of grade C post-hepatectomy liver dysfunction, defined by the International Study Group of Liver Surgery, and the 30-day PHLF-related mortality [32,33]. Combined scores ranged from approximately −4 to 2, with higher scores correlating with worse outcomes [32].

The different clinical risk scores are summarized in Table 1. While each of these clinical risk predictors offers valuable information for clinical decision-making, challenges persist as most of these models were originally designed for different purposes. Only the ALBI score was specifically developed to predict risk in patients undergoing hepatectomy for HCC [30]. Although the VOCAL-Penn score aimed to address the lack of surgery-type stratification in previous models like the CTP, MELD, and MRS, the operative categories in this scoring system remained broad and did not include liver resection [27]. Moreover, the CTP classification was neither developed nor validated in patients with HCC, and one of its major drawbacks was the subjectivity of the clinical variables [21]. Furthermore, postoperative mortality after hepatic resection has declined over the last two decades likely due to advances in treatment, technology, and improvement in the care of critically ill patients [18]. As a result, some studies have indicated that earlier risk models are overpredicting postoperative mortality risk.

## 5. Preoperative Assessment–Imaging Modalities and Measurement of Hepatic Reserve

The assessment of hepatic reserve is crucial when evaluating a patient for potential liver resection, especially in the presence of parenchymal disease. The extent of the resection and function of the future liver remnant (FLR) are the most significant predictors of post-hepatectomy liver failure [34]. For example, patients with healthy liver parenchyma can typically tolerate a resection resulting in a 25% FLR, whereas those with damaged liver parenchyma require a 40% FLR [35]. When evaluating a cirrhotic patient for liver surgery, it is crucial to consider both the functional capacity of the FLR and the volume. Noninvasive tests such as cross-sectional imaging, elastography, and nuclear medicine imaging serve as valuable tools for assessing liver volume, parenchymal quality, and functional capacity. Table 2 and Table 3 summarize the imaging modalities used to assess the structural features and functional capacity of the liver, respectively.

### 5.1. Ultrasound (US) and Ultrasound-Based Elastography

#### 5.1.1. Conventional US

US is often used as the initial modality for assessment of the liver in patients with suspected liver disease due to its easy accessibility, low cost, and lack of ionizing radiation. Findings on US that are suggestive of fibrosis include heterogenous echogenicity of the parenchyma and surface nodularity. As the fibrosis worsens and cirrhosis develops, the caudate lobe will appear hypertrophied and may be associated with a partial volume decrease in the right side [36]. Doppler assessment of the portal vein diameter, velocity, and direction of flow allows the detection of portal hypertension [36].

#### 5.1.2. US-Based Elastography

Elastography techniques use mechanical excitation of the hepatic parenchyma while monitoring the response. There are two primary types of US-based elastography commonly used to evaluate liver stiffness and fibrosis.

Strain elastography (SE): There are two types of SE that differ by the mechanism in which the strain is generated. Stress is applied to the tissue, either by manual compression or through physiologic movement (i.e., pulsation, breathing), and the tissue displacement is measured as a relative representation of elasticity [37].

Shear wave elastography (SWE): Shear waves are generated from acoustic radiation force impulse (ARFI) and transducer-derived mechanical pulse, and the speed at which they propagate through tissue is measured [36,37]. The speed is a qualitative and quantitative representation of the elasticity of the tissue it is traversing as the shear waves propagate faster in fibrotic tissue [37]. There are three methods for performing SWE and differ in the mechanism by which the shear wave is generated: 1-dimensional transient elastography (1D-TE), point shear wave elastography (pSWE), and 2D-SWE [37].

A FibroScan is a commonly used 1D-TE and has been shown to accurately diagnose cirrhosis based on stiffness cutoff values, measured in kilopascals (kPa): significant fibrosis (F2, 7.5 kPa to 10 kPa), severe fibrosis (F3, 10 kPa to 14 kPa), and cirrhosis (F4, > 14 kPa) [38]. It can also distinguish significant fibrosis from non-significant (F0 and F1) but cannot discriminate between individual fibrosis stages [37,38]. Studies have attempted to identify a cut-off value (kPa) that predicts PHLF, but due to the heterogeneity between the groups, no single value has been validated, and there is a large range of values proposed by different studies [38,39,40,41].

### 5.2. Computed Tomography (CT) and Volumetry

CT is an insensitive tool in detecting early cirrhosis [36]. Early indicators of a diseased background parenchyma are heterogeneity and hypoattenuation in comparison to the spleen. With more advanced disease, CT findings may include a nodular appearance of the liver, a liver with rounded edges, splenomegaly, or portosystemic venous collaterals [36]. One study assessing the capacity of CT for predicting fibrosis in patients with hepatitis C demonstrated that when CT data are combined with laboratory-based measures (FIB-4 score and aspartate transaminase-to-platelet ratio index [APRI]), the diagnostic accuracy is similar to transient elastography [42,43]. The CT-based parameters included liver surface nodularity score, texture analysis, hepatic and splenic volumetric analysis, and fissural widening [42].

### 5.3. Magnetic Resonance Imaging (MRI)

#### 5.3.1. Conventional MRI

Similarly, the use of MRI is insufficient in detecting the more subtle changes in the earlier stages of fibrosis. A liver MRI performed on a patient with diffuse nonalcoholic fatty liver disease (NAFLD) will demonstrate reduced signal intensity on the opposed-phase T1-weighted images [44]. Regional differences in perfusion may suggest segmental distribution of fat infiltration [44]. A fibrotic liver will have peak enhancement in later phases compared to healthy liver parenchyma. In more advanced disease, structural changes, such as surface nodularity, hypertrophy of the caudate, regenerative nodules, or evidence of portal hypertension (portosystemic venous collaterals, splenomegaly, and ascites), are seen [36].

#### 5.3.2. MRI Elastography (MRE)

MRE uses modified phase-contrast imaging sequences to detect shear waves propagated through the liver by way of a passive driver placed against the patient’s right anterior chest wall [45]. Vibrations are conducted into the body, which produces mechanical waves, and images are obtained with fast pulse sequences, measuring the speed of the shear waves through the liver. Elastograms are generated, and a quantitative stiffness measurement is obtained [43]. MRE is sensitive to the detection of mild, significant, and severe fibrosis (77%, 87%, and 89%, respectively) [46]. In a systematic review and pooled analysis, similar findings were observed with an area under the receiver operating characteristics curve (AUROC) values of 0.89, 0.93, 0.93, and 0.95 in detecting any (≥F1), significant (≥F2), or severe (≥F3) fibrosis and cirrhosis (F4), respectively [43,45]. When compared to TE, MRE has the advantage of assessing the entire liver, is more accurate, and is not limited in obese patients, those with ascites, or who lack an acoustic window [36,47].

### 5.4. Functional Assessment

#### 5.4.1. Indocyanine Green Retention Rate

Indocyanine green (ICG) is a water-soluble dye that is administered intravenously and taken up exclusively by hepatocytes. It is then excreted into the biliary system without undergoing any modification [1]. The clearance of ICG is dependent on the function of hepatocytes, biliary excretion, and blood flow [48]. The percentage of ICG retained at 15 min (ICG R15) is a marker of hepatic function, with a normal retention rate being <10% [48]. The ICG clearance test is widely used in Eastern countries as a liver function test [49,50]. In patients with compromised liver function, higher ICG R15 values are observed, indicating impaired hepatic function [48]. This impairment is notably due to decreased hepatic blood flow and diminished uptake from sinusoids into hepatocytes because of cirrhosis [51]. ICG retention can also be measured to detect advanced liver fibrosis and cirrhosis with a high predictive value [52].

Imamura et al. proposed a decisional algorithm for selecting an operative procedure in patients with impaired liver function using ICG retention at 15 min, where retention of < 20% allowed for major hepatic resection [51]. Studies have shown that ICG R15 can predict postoperative liver failure and mortality, making it an invaluable tool in surgical decision-making [53]. Furthermore, ICG clearance is used postoperatively to monitor liver function and detect early signs of liver failure, guiding postoperative management and interventions to mitigate complications [53]. While the ICG clearance test offers a non-invasive, functional assessment of the liver and can stage liver fibrosis, it measures global liver function, can be affected by vascular and biliary obstruction, and has not been widely adopted in Western countries [49,50,52].

#### 5.4.2. Liver Maximum Capacity (LiMAx) Test

The LiMAx test is a bedside test that evaluates maximal liver function capacity through the assessment of ^13^C-methacetin kinetics. ^13^C-methacetin is a synthetic substrate that is metabolized exclusively by hepatocytes through the action of the cytochrome P450 enzyme CYP1A2 into ^13^CO2 and paracetamol [54]. The test is performed by administering intravenous ^13^C-methacetin and subsequently measuring ^13^CO2/^12^CO2 in the exhaled air, which represents the functional capacity of the liver [54]. It is a dynamic study that estimates the risk of PHLF by quantifying the enzymatic activity in real-time, providing insight into the liver function and its ability to recover and regenerate after major hepatectomy [54].

While this test may be useful in predicting PHLF, it does not fully capture the multifaceted aspects of liver disease, such as fibrosis [55,56]. As such, more recent studies have highlighted the limitations of the LiMAx test, including its insensitivity to changes in liver function related to treatments like chemotherapy or portal vein embolization [56]. The liver undergoes physiologic alterations after these interventions, which may not necessarily affect CYP1A2 enzyme activity and, therefore, will not be measured by the LiMAx test [56]. Additionally, compared to APRI-ALBI, which is derived from routine lab tests and offers advantages in terms of cost and availability, the LiMAx test is inferior in forecasting PHLF and associated mortality [55].

#### 5.4.3. ^99m^Tc-Labeled Galactosyl-Human Serum Albumin Scintigraphy (^99m^Tc-GSA)

^99m^Tc-GSA single photon emission computed tomography (SPECT) was developed to visualize and quantify binding to the asialoglycoprotein receptor, which is present only in hepatocytes, as a measurement of hepatic function [57]. ^99m^Tc-GSA binds to the surface receptor on the hepatocyte, enters the cell through endocytosis, and gets degraded. The hepatic uptake ratio and blood clearance ratio of ^99m^Tc-GSA are the most frequently used parameters obtained from ^99m^Tc-GSA scintigraphy and are an accurate representation of hepatic function. Because ^99m^Tc-GSA is not excreted in the biliary system and is not altered by hyperbilirubinemia, it remains a reliable predictor of hepatic function in compromised livers. Additionally, both dynamic and static ^99m^Tc-GSA SPECT are used to measure overall functional volume and segmental liver function, providing both a visual and quantitative assessment of total and regional liver function, which accounts for the heterogeneous distribution of functional mass in compromised livers [57,58]. Studies have shown that as the degree of fibrosis increases, the uptake ratio decreases, and more postoperative complications and PHLF were seen in patients with lower hepatic clearance of ^99m^Tc-GSA [59]. 

#### 5.4.4. ^99m^Tc-Labeled Mebrofenin Hepatobiliary Scintigraphy (HBS)

Mebrofenin is an amino diacetic acid (IDA) that enters hepatocytes and is excreted into the biliary system without undergoing biotransformation [58]. ^99m^Tc-mebrofenin HBS is an ideal agent to measure liver function since it has high hepatic uptake, low urinary excretion, and strong resistance to displacement by hyperbilirubinemia [57]. The ^99m^Tc-mebrofenin HBS extraction rate is correlated to underlying parenchymal status and has been validated for use preoperatively to predict the risk of PHLF if the uptake is below 2.69%/min/m^2^ [60]. Similarly to ^99m^Tc-GSA, both a visual and quantitative assessment of total and regional liver function are provided, making its use more valuable than CT volumetry for the prediction of PHLF [57].

The comprehensive assessment of hepatic reserve is of paramount importance in the preoperative evaluation for liver resection. This evaluation is not only pivotal for determining the feasibility of surgery but also for planning the extent of resection while ensuring a viable and functional FLR. These noninvasive imaging modalities play a critical role in this multifaceted assessment, offering valuable insights into liver volume, structural integrity of the parenchyma, and functional capacity of the liver. The benefit of integrating multiple imaging modalities is that nuclear imaging studies such as ^99m^Tc-GSA and ^99m^Tc-mebrofenin HBS assess the function of the FLR and can accurately predict PHLF in both healthy and diseased livers [57]. A recent study evaluating the relationship between liver stiffness measured by TE and liver function measured by ^99m^Tc-mebrofenin HBS demonstrated a decrease in mebrofenin uptake with increasing stiffness (Pearson r = −0.634, *p* = 0.001) [61]. This provides insight beyond a single variable like volumetry or degree of fibrosis and provides a comprehensive, dynamic view of the liver’s functional landscape.

## 6. Preoperative Optimization to Reduce Postoperative Complications of Cirrhosis

### 6.1. Metabolic Alterations and Protein-Calorie Malnutrition

Patients with cirrhosis have multiple metabolic alterations due to the liver’s role in the production, storage, and metabolism of essential nutrients [62]. Protein-calorie malnutrition is reported in up to 90% of cirrhotic patients, resulting in sarcopenia due to high resting energy expenditure, impaired hepatic protein synthesis, malabsorption, and poor dietary intake [8,62]. This results in a myriad of postoperative complications, including surgical site or deep space infection, impaired wound healing, wound dehiscence, accumulation of ascites, impaired hepatic regenerative response, and death [8].

Preoperatively, patients should undergo a comprehensive nutritional assessment, which includes an evaluation of muscle mass, global assessment tools such as the Royal Free Hospital-Nutritional Prioritizing Tool (RFH-NPT), and a detailed nutritional intake to determine the degree of malnutrition [63]. Albumin and prealbumin should be measured as low levels are predictive of poor surgical outcomes. To optimize the malnourished cirrhotic patient, daily caloric intake should be 35 kcal/kg/day, protein intake should be 1.5 g/kg/day, and supplementation with vitamins A, D, E, C, and K should be administered [63,64].

The European Society for Clinical Nutrition and Metabolism (ESPEN) recommends nutritional support of at least 7–14 days preoperatively in mildly malnourished patients and a longer period of nutritional supplementation for individuals with severe malnutrition to reduce short-term mortality and postoperative complications [65,66]. Taste can be a significant barrier to patient compliance with a high-protein diet. Patient education regarding the benefits and possible alternatives, such as flavor enhancements or different formulations, can help improve compliance [66]. In cases where oral intake is deemed insufficient, enteral nutrition or parenteral nutrition should be considered, particularly for severely malnourished patients [66].

### 6.2. Altered Coagulation

Patients with severe fibrosis or cirrhosis often experience significant alterations in their hemostatic system due to the liver’s role as the primary site for the synthesis of both coagulation proteins and their inhibitors [67]. This imbalance can lead to episodes of both bleeding and thrombosis [67]. In the context of preoperative optimization, the correction of thrombocytopenia and other abnormal laboratory measures of coagulation remains debated. According to the European Association for the Study of the Liver (EASL) clinical practice guidelines, there is no strong evidence supporting the routine correction of thrombocytopenia or the infusion of thrombopoietin receptor agonists to prevent procedure-associated bleeding if the platelet count is above 20–50 × 10^9^/L but should be considered in patients undergoing high-risk procedures when the platelet count is < 20 × 10^9^/L [68]. Moreover, the EASL recommends against the routine correction of an abnormal INR using blood products or factor concentrates due to the negatively associated risks, such as increasing blood volume and thereby portal pressures [68]. Likewise, patients with portal hypertension-related bleeds should be managed with portal hypertension-lowering measures [68].

### 6.3. Portal Hypertension, Varices, and Ascites

PH has classically been considered a relative contraindication for liver resection in patients with HCC due to the reported risk of postoperative morbidity, PHLF, and mortality [11]. When HVPG is not available, clinical findings suggestive of PH, including esophageal varices or a platelet count < 100,000/mL with splenomegaly, are used as surrogates [11,69,70]. The presence of PH adds complexity to the perioperative management in patients undergoing liver surgery. First, the administration of fluids for resuscitation to avoid hypotension and resultant hepatic ischemia may increase portal pressure and lead to variceal bleeding [8,17]. Second, a shift in the fluid balance exacerbating portal hypertension may lead to ascites production and increased risk of postoperative renal failure [17].

Patients with ascites should be optimized with medical therapy, including a sodium-restricted diet and diuretics. Additionally, an endoscopy should be performed, and high-risk varices should be treated to reduce the risk of perioperative bleeding [71]. The addition of a nonselective beta-blocker should also be considered to help reduce portal pressure and the risk of variceal bleeding [72]. Terlipressin, a vasopressin analog, has shown efficacy in decreasing intraoperative portal pressure, blood loss, and the number of blood transfusions [73]. It has also been shown to decrease postoperative portal pressure in cirrhotic patients undergoing liver resection [73]. Despite this, there is not enough evidence supporting the preoperative use of Terlipressin for the prevention of complications in patients undergoing liver resection [73]. TIPS also has a recognized role in the prevention and treatment of complications from portal hypertension, including variceal bleeding and refractory ascites [74]. There are several small case series that suggest performing TIPS in the preoperative setting in patients with portal hypertension reduces the risk of bleeding, ascites accumulation, and postoperative liver failure [71,74,75].

### 6.4. Portal Flow Modulation Strategies

Portal flow modulation is a surgical technique used to optimize blood flow to the liver after hepatic resection. It involves adjusting the hepatic artery and portal vein inflow to ensure optimal perfusion to the liver remnant to prevent irreversible sinusoidal injury from high portal vein pressures [76]. This concept was initially applied in living donor liver transplantation but has shown positive outcomes when used in patients with cirrhosis [76,77,78]. Commonly employed techniques to accomplish portal flow modulation include portal flow diversion, splenectomy, and splenic artery ligation [76].

In a systematic review of eight studies with 1445 patients with HCC and CSPH, hepatectomy vs. hepatectomy plus splenectomy was compared [79]. The authors noted a significantly improved 5-year survival in the hepatectomy plus splenectomy group compared to the hepatectomy alone, with no difference in blood loss, transfusion, postoperative complications, or mortality noted [79]. Another study evaluated the outcomes in patients with HCC and hypersplenism depending on whether splenectomy was performed prior to hepatectomy or at the time of hepatectomy [80]. The authors found that regardless of the timing of splenectomy, DFS was improved compared to the hepatectomy alone group and did not add increased surgical risk [80]. A study assessing concurrent splenectomy and esophagogastric devascularization in patients with HCC and CSPH undergoing liver resection demonstrated improved overall survival and decreased postoperative complications compared to the patients who underwent liver resection alone [81].

## 7. Discussion

This review underscores the potential for expanding liver resection criteria beyond the BCLC guidelines, highlighting the importance of comprehensive preoperative assessments and predictive risk management. The pathophysiology of chronic liver disease, cirrhosis, and portal hypertension elevates the risk of surgical morbidity and mortality. These patients are at increased risk of surgical bleeding due to complications from portal hypertension, and coagulation disturbances further heighten the risk of bleeding or thrombosis. Additionally, these patients experience sarcopenia due to dysfunction in protein synthesis, leading to impaired wound healing [17]. Consequently, elective surgery is typically avoided in patients with cirrhosis or evidence of portal hypertension. The problem, however, is that HCC typically arises in the background of cirrhosis and is the primary indication for hepatic resection in cirrhotic patients [82]. Advancements in surgical techniques and perioperative care, as demonstrated by improved outcomes in high-risk patients, challenge the traditional risk models which may now overpredict mortality rates. In pursuit of enhancing survival prospects for such “nonideal” patients, several studies have successfully deviated from the stringent BCLC guidelines with acceptable short- and long-term outcomes [83,84].

Clinical findings suggestive of PH, such as esophageal varices, splenomegaly, and thrombocytopenia, are commonly used as surrogates for HVPG. While an increased HVPG is associated with postoperative liver dysfunction and mortality after liver resection and correlates linearly with liver stiffness, indirect criteria of PH were not found to have the same association [12]. Likewise, more than 50% of patients with compensated cirrhosis with HVPG > 10 mmHg may have no varices and a normal platelet count [70]. Furthermore, findings from studies evaluating surgical outcomes in patients with noncirrhotic portal hypertension suggest that clinical manifestations of PH can be present even with a normal or mildly elevated HVPG [85]. These insights are crucial as they suggest a paradigm shift in preoperative evaluation from relying solely on indirect markers to incorporating direct HVPG measurements to better stratify which patients can safely undergo liver resection.

One drawback of the HVPG measurement is its invasiveness. In a systematic review encompassing eight studies, the utility of performing transient elastography to measure liver stiffness (kPa) compared to HVPG was evaluated [86]. There was a moderate to high correlation between liver stiffness and HVPG (r = 0.552–0.86), which was especially prominent in patients with milder forms of chronic liver disease. However, the predictive capacity of liver stiffness for PH was limited as the severity of liver disease and PH increased [86]. Further, liver stiffness values of 17.6–23 kPa were associated with a HVPG ≥ 12 mmHg [86]. Discrepancies were observed in liver stiffness cutoff values indicative of fibrosis (12.6 kPa) and CSPH (19.6 kPa) as well as for predicting PHLF in cirrhotic (17.6 kPa) versus noncirrhotic (15.7 kPa) patients [39]. Another study identified a liver stiffness cutoff value of 12 kPa as a risk factor for major postoperative complications, increased operative blood loss, and blood transfusion requirements [41]. Interestingly, findings of cirrhosis on preoperative imaging or intraoperative assessment were not as sensitive as liver stiffness measurements in predicting postoperative complications [41]. These studies highlight the clinical significance of noninvasive liver stiffness measurements, revealing that a liver stiffness of 12 kPa is associated with increased postoperative complications compared to using a CT diagnosis of cirrhosis alone. Moreover, higher liver stiffness values correlate with CSPH, indicating that the degree of fibrosis poses a higher surgical risk even before CSPH manifests.

**Figure 2:**
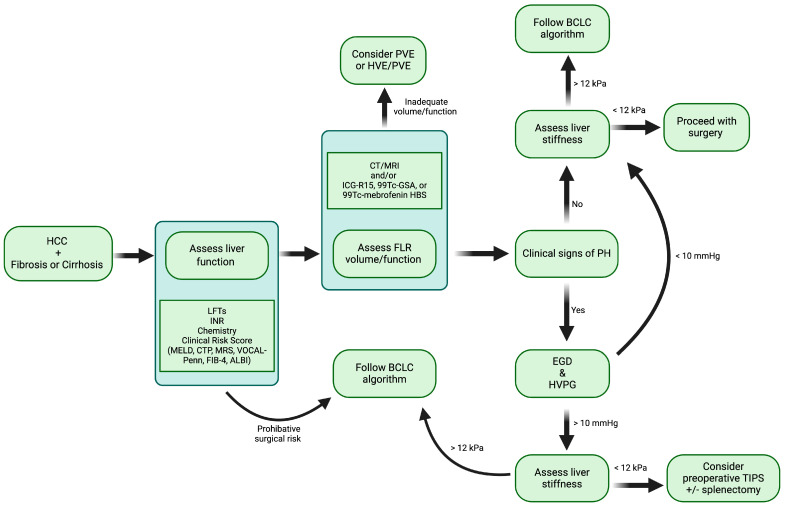
Summary of preoperative optimization and decision-making in patients with resectable HCC and cirrhosis. HCC, hepatocellular carcinoma; LFTs, liver function tests, INR, international normalized ratio; MELD, model for end-stage liver disease; CTP, Child-Turcotte-Pugh; MRS, Mayo risk score; VOCAL-Penn, Veterans outcomes and costs associated with liver disease; FIB-4, Fibrosis-4; ALBI, albumin-bilirubin score; FLR, future liver remnant; CT, computed tomography; MRI, magnetic resonance imaging; ICG-R15, indocyanine green retention rate; ^99m^Tc-GSA, ^99m^Tc-labeled galactosyl-human serum albumin scintigraphy; ^99m^Tc-mebrofenin HBS, ^99m^Tc-labeled mebrofenin hepatobiliary scintigraphy; PVE, portal vein embolization; HVE, hepatic vein embolization; BCLC, Barcelona Clinic Liver Cancer; PH, portal hypertension; EGD, esophagogastroduodenoscopy; HVPG, hepatic vein portal gradient; TIPS, transjugular intrahepatic portosystemic shunt; kPa, kilopascal; mmHg, millimeter of mercury.

The success of liver resection for HCC in patients who fall outside the traditional BCLC criteria hinges on a comprehensive approach to patient selection and preoperative evaluation. The key selection criteria should include the severity and control of portal hypertension, extent of liver fibrosis, overall liver function, nutritional status, and the size, number, and location of liver tumors [70,75,80,83]. To effectively assess these factors, several preoperative tests are recommended to improve patient selection. Utilizing a clinical risk score to predict surgical risk serves as an initial step in this process. Evaluating portal hypertension severity through HVPG or liver stiffness with transient elastography aids in predicting adverse postoperative outcomes. Obtaining this information directly has demonstrated greater accuracy compared to using indirect measures of CSPH. For cases involving CSPH, preoperative placement of a TIPS can significantly enhance surgical outcomes [75]. Preoperative TIPS has been shown to lower the incidence of acute liver failure, reduce postoperative ascites and transfusion needs, decrease postoperative mortality rates, and improve 1-year survival outcomes while effectively mitigating the effects of CSPH [75]. In patients exhibiting CSPH with an HVPG ≥ 10 mmHg yet displaying satisfactory surgical candidacy—characterized by low ASA scores, well-preserved liver function, and favorable tumor biology—postoperative mortality, morbidity, and rates of liver decompensation were found to be acceptable [70]. The 1-, 3-, and 5-year overall survival rates for patients undergoing liver resection for HCC with CSPH were 89%, 73%, and 55%, respectively [70]. In contrast, the median survival for similar patients receiving the best nonoperative management as recommended by BCLC criteria is less than 36 months [70,87]. Obtaining information regarding the quality of the liver parenchyma and detailed tumor anatomy with enhanced imaging techniques such as MRI or CT, along with gathering a functional assessment of the liver with nuclear medicine imaging modalities, allows for more precise and informed surgical planning. This complete imaging approach ensures an optimal patient selection and the tailoring of surgical strategy (Figure 2).

Incorporating these comprehensive diagnostic tools provides a more accurate assessment of surgical risks and enables surgeons to tailor their approaches to each patient’s specific condition. Thus, liver resection, previously deemed too risky for certain patients with HCC, should be considered a viable option under the ideal circumstances in “nonideal” patients. The integration of these tools advocates for a more flexible approach to the BCLC guidelines. Such an inclusive approach may improve overall survival in select patients with HCC by extending potentially curative treatment to a wider cohort of patients.

## Figures and Tables

**Table 1:** Preoperative surgical risk models for patients with cirrhosis.

**A.** Scores used for predicting mortality in general surgery.				
**Preoperative Risk Model**	**Variables**	**Original Function**	**Group/Score**	**Reported Outcome**
CTP	Ascites Encephalopathy INR Albumin Bilirubin	Guide selection of patients for portosystemic shunt. Predict mortality in cirrhotic patients.	Class A (5–6 points)	Survival: >15 years Perioperative mortality: 10%
			Class B (7–9 points)	Survival: Transplant referral Perioperative mortality: 30%
			Class C (10–15 points)	Survival: 1–3 years Perioperative mortality: 82%
MELD	INR Serum creatinine Serum bilirubin	Predict 30-day mortality after TIPS	≤9 10–19 20–29 30–39 ≥40	1.9% 6% 19.6% 52.6% 71.3%
MRS	MELD ASA class Age	Predict short- and long-term mortality after elective surgery in cirrhotic patients	Calculated score	Mortality prediction varies based on combined variables
VOCAL-Penn Model	Age Serum albumin Serum bilirubin Platelet count BMI NAFLD ASA class Surgery type	Predict postoperative 30-, 90-, 180-day mortality after specific operations	Calculated score	Mortality prediction based on surgery type and combined variables
**B.** Scores used for predicting fibrosis stage.				
**Fibrosis Score Model**	**Variables**	**Original Function**	**Score**	**Reported Outcome**
FIB-4	Age AST ALT Platelet count	Assess liver fibrosis in patients with NAFLD	<1.45 1.45–3.25 >3.25	Fibrosis stage: 0–1 2–3 4–6
Forns Score	Age GGT Cholesterol Platelet count	Identify liver fibrosis in chronic hepatitis C	<4.21 >6.9	Absence significant fibrosis Presence significant fibrosis
**C.** Scores used for predicting mortality after liver surgery.				
**Preoperative Risk Model**	**Variables**	**Original Function**	**Score**	**Reported Outcome**
ALBI	Serum bilirubin Serum albumin	Predict overall survival after hepatectomy for HCC	≤−2.6 >−2.6 to ≤−1.39 >−1.39	Median Survival 18.5–85.6 months 5.3–46.5 months 2.3–15.5 months
APRI + ALBI	Serum bilirubin Serum albumin AST Platelet count	Predict post-hepatectomy liver dysfunction and associated 30-day mortality	Calculated score	Post-hepatectomy liver dysfunction and associated 30-day mortality

CTP, Child–Turcotte–Pugh; MELD, model for end-stage liver disease; MRS, Mayo risk score; VOCAL-Penn, Veterans outcomes and costs associated with liver disease; FIB-4, Fibrosis-4; ALBI, albumin-bilirubin score; INR, international normalized ratio; ASA, American society of anesthesiologists; BMI, body mass index; NAFLD, non-alcoholic fatty liver disease; AST, aspartate aminotransferase; ALT, alanine aminotransferase; GGT, gamma-glutamyl transferase; APRI, aspartate aminotransferase/platelet ratio index; TIPS, transjugular intrahepatic portosystemic shunt; HCC, hepatocellular carcinoma.

**Table 2:** Imaging tests for structural evaluation of the liver in patients with liver fibrosis or cirrhosis.

Imaging Modality	Primary Use	Key Features and Indications for Use	Advantages	Limitations
Ultrasound (US)	Initial assessment of liver	Detects liver fibrosis indicators and nodularity Doppler evaluation	Accessible Cost-effective Non-ionizing Useful for basic structural and flow assessment	Operator dependent Limited sensitivity in early fibrosis detection
US-based transient elastography	Evaluating liver stiffness and fibrosis	Includes strain elastography and shear wave elastography Measures tissue elasticity	Non-invasive Provides qualitative and quantitative data on liver stiffness	Limited in obesity and ascites Does not distinguish between fibrosis stage
Computed Tomography (CT)	Detailed liver anatomy imaging	Assesses liver surface nodularity, texture, spleen size Visualization of varices Early cirrhosis detection is limited	More detailed than ultrasound Used for characterization and surgical planning Volumetric analysis	Exposure to ionizing radiation Less sensitive in detecting early fibrosis
Magnetic Resonance Imaging (MRI)	Advanced imaging for liver anatomy	High-resolution images of liver anatomy and pathology Detects regional fat infiltration and fibrosis	Superior for detailed parenchymal evaluation Useful in advanced fibrosis and cirrhosis	High cost Long examination time Limited availability
MRI Elastography (MRE)	Assessing liver stiffness and fibrosis	Uses shear waves to generate elastograms for stiffness measurements Highly sensitive to fibrosis	Accurate across all stages of liver disease Not limited by obesity or ascites	High cost Long examination time Limited availability

**Table 3:** Imaging tests for liver functional assessment.

Imaging Modality	Primary Use	Key Features	Indications and Advantages
Indocyanine green retention rate (ICG R15)	Functional assessment of the liver	Measures the clearance of ICG dye as an indicator of liver function	Provides dynamic information on liver function Useful in surgical planning
^99m^Tc-labeled galactosyl-human serum albumin scintigraphy (^99m^Tc-GSA)	Quantifying liver function	Measures binding to asialoglycoprotein receptors in hepatocytes	Offers both visual and quantitative data on liver function Useful in assessing FLR function
^99m^Tc-labeled mebrofenin hepatobiliary scintigraphy (HBS)	Measuring liver function for preoperative assessment	Assesses hepatocyte function through biliary excretion High hepatic uptake and resistance to bilirubin interference	Ideal for predicting PHLF Provides detailed functional mapping of the liver More valuable than CT volumetry for PHLF prediction

FLR, future liver remnant; PHLF, post-hepatectomy liver failure.
